# Variability of joint hypermobility in children: a meta-analytic approach to set cut-off scores

**DOI:** 10.1007/s00431-024-05621-4

**Published:** 2024-05-27

**Authors:** Cylie M. Williams, James J. Welch, Mark Scheper, Louise Tofts, Verity Pacey

**Affiliations:** 1 School of Primary and Allied Health Care, Monash University, 47-49 Moorooduc Hwy, Frankston, VIC 3199 Australia; 2 Ablefeet, 16 Terrace Road, Walton-on-Thames, Surrey, KT12 2SB UK; 3 Research Center Innovations in Care, Data Supported Healthcare, Rotterdam University of Applied Sciences, Rotterdam, The Netherlands; 4 Department of Health Sciences, Faculty of Medicine, Health & Human Sciences, Macquarie University, North Ryde, NSW 2113 Australia; 5 Institute for Communication, Media and Information Technology, Program Responsible AI, Rotterdam University of Applied Sciences Rotterdam, Rotterdam, The Netherlands; 6 Livinglab Responsible AI, Creating010, Rotterdam University of Applied Sciences Rotterdam, Rotterdam, The Netherlands

**Keywords:** Beighton score, Hypermobility, Ehlers Danlos, Child, Adolescent

## Abstract

Current international consensus of the appropriate Beighton score cut-off to define if a child has generalised joint hypermobile or not is based upon expert opinion. Our aim was to determine the prevalence of Beighton scores of children worldwide to provide a recommendation for establishing the Beighton score cut-off to identify generalised joint hypermobility in children. We used AMED, OVID Medline, Embase and CINAHL to find published articles from inception to April 2024 describing Beighton scores of children up to and including 18 years from the general population. We extracted study demographics including country of publication, total number of participants, summary data about the age and sex of participant, Beighton scores and any cut-off used where authors deemed children hypermobile and how many children were rated at the corresponding Beighton scores. There were 37 articles reporting on the prevalence or incidence of hypermobility at cut-off scores from 28,868 participants. Using the cut-off of ≥ 6 resulted in a prevalence of 6% for studies reporting male data and 13% for studies reporting female data. Limited data reporting availability precluded further sub-analysis at a Beighton score of ≥ 7, age, pubertal status and ethnicity.

*    Conclusion*: The working threshold for identifying generalised joint hypermobility in children should be a Beighton score of 6 or more. Our analysis also suggests a Beighton score of 7 or greater may be appropriate in childhood, particularly for females.

**Table:** 

**What is Known:** *• The working threshold for identifying generalised joint hypermobility in children previously was set based on expert opinion.*	
**What is New:** *• The threshold to identify hypermobility in children should be at a minimum of ≥ 6 on the Beighton score.*

**Supplementary Information:**

The online version contains supplementary material available at 10.1007/s00431-024-05621-4.

## Introduction

Joint hypermobility, the movement of multiple joints beyond what is considered typical range of motion, is a commonly reported physical trait of childhood [1]. The prevalence of joint hypermobility varies markedly in children across studies, hypothesised to be related to age [1], pubertal status [2], sex [3] and ethnicity [4]. The most commonly used screening tool to identify generalised joint hypermobility is the Beighton score which has acceptable reliability [5]. The Beighton score is quick to perform and is well known internationally. This allows for ease of use as a screening tool in global settings, despite the tool’s known limitations being upper limb dominant and including combined joint movements [6]. The Beighton score is a continuous scale out of 9; however, it is commonly accepted that a cut-off score serves as a dichotomous indicator of whether a child has generalised joint hypermobility or not.

The accepted standard within paediatric orthopaedics health care identifies children’s measurable physical traits falling outside of two standard deviations from the population mean, presenting as outside of typical variance [7]. This identifies children in the upper and lower 2.5% of the population as presenting atypically from the remaining 95% of the population. Defining typical variance with different screening or assessment tools allows clinicians to determine atypical traits and guides the most appropriate clinical course of care when required [7].

Current international consensus of the appropriate cut-off score of the Beighton to define if a child is hypermobile or not, is based upon expert clinical consensus, rather than these accepted standards [8, 9]. Consequently, a cut-off score indicating generalised joint hypermobility should be positioned at two standard deviations above the population mean to fit within accepted clinical conventions. This approach allows a robust approach to identifying the appropriate cut-off Beighton score indicating when a child presents with generalised joint hypermobility [10]. In the absence of comprehensive normative data using this approach, the recent diagnostic framework for paediatric hypermobility disorders recommended a cut-off score indicating generalised joint hypermobility at 6/9 for all children and adolescents up to 18 years of age and prior to skeletal maturity [8].

Therefore, this present study aimed to determine the prevalence of Beighton scores of children worldwide to provide a recommendation for establishing the Beighton score cut-off to identify generalised joint hypermobility in children. Where possible, this present study aimed to consider the impact of age, sex, pubertal status and ethnicity on the prevalence of generalised joint hypermobility in children and adolescents.

## Methodology

Data were collated through a systematic review performed and reported in accordance with the Preferred Reporting Items for Systematic Reviews and Meta-Analyses (PRISMA) guidelines checklist (Supplementary File 1) [11]. The review protocol was prospectively registered on PROSPERO (CRD42021248465). The study question was developed using the PICO (Population, Intervention, Comparison and Outcomes) model [12]. The review keywords were targeted to the purpose of this review. The search terms included Beighton Score/scale, hypermobility, Ehlers Danlos syndrome, children and adolescents (Supplementary File 2: Search strategies).

An electronic search was performed in AMED (Allied and Complementary Medicine), OVID Medline, Embase and CINAHL from inception to the 18th of April, 2024, by the primary author. Covidence systematic review software (Veritas Health Innovation) was utilised to screen and manage articles. Titles and abstracts were screened independently by two independent team members (CMW and JJW) and conflicts resolved by a third team member (VP) against the inclusion and exclusion criteria. Articles were included only when describing Beighton scores of children up to and including 18 years from the general population. Articles were excluded if the full text was not in English, if children described musculoskeletal pain, if the population was recruited within a health care service or clinic assessing or treating conditions relating to hypermobility or from a cohort of children where hypermobility may lead to successful sporting participation (e.g. dancing or gymnastics). Where data were grouped with young people aged 19 or above, we included data where the reported cohort mean age was 18 years or less, or we removed any data where the participants were aged 19 years or older from the analysis if it was reported separately. If we were unable to extract data from children older than 18 years, we excluded all data. If studies of children with known conditions with joint hypermobility and the study reported a comparable community-based comparison or control group, data from the only control group were extracted.

All full-text included articles were independently screened by two reviewers (CMW and JJW), and again, any disagreements were discussed and resolved with a third reviewer (VP or MS). The authors of studies were contacted about unpublished data to support inclusion when the article provided limited information about the cohort, or Beighton score groupings were limited. If these authors did not respond or were unable to provide meaningful data to support inclusion, articles were excluded. We used forward and backward chaining methodology to check references and citations of included articles to ensure as many articles were included as possible.

### Data extraction and quality assessment

A custom data extraction template was developed in Microsoft Excel (Microsoft Corporation, 2023). One reviewer independently extracted article data (CMW or JJW), and this was cross checked by a second reviewer (CMW or JJW). Where there were any differences, these were highlighted and discussed with the additional third reviewer for confirmation (VP). Data extraction from each article included study characteristics such as the country of publication, total number of participants, summary data about the age and sex of participant, Beighton scores and any cut-off score authors deemed children as having joint hypermobility, profession of assessors and how many children were rated at the corresponding Beighton Score/s. Due to variability in how ethnicity was reported, we used world region as a proxy for ethnicity. We also pre-planned to extract pubertal status according to age from studies; however, this was also inconsistently reported; therefore, no puberty status data were retained in the final extraction template.

We planned to assess the risk of bias using the ROBINS-I tool. We deviated from the registered protocol and used the JBI Critical Appraisal Checklist for Studies Reporting Prevalence Data [13]. This deviation was due to the checklist being more appropriate to describe the risks of bias in the included study designs. Each of the quality indicators were scored with a Yes, No, Unclear or Not applicable. Questions included responding to the study’s sample frame, appropriateness of sample, sample size, setting description, data analysis, description of condition (or absence of), measurements, statistical analysis, and response rate.

### Data analysis

Microsoft Excel (Microsoft Corporation, 2022) was used to collate data extracted from the included manuscript in terms of author, year of publication, age (mean/median, standard deviation (SD) or interquartile range (IQR), range), country, population (total, sex specific totals) and Beighton Scores for population (number of positives/negatives relating to author determined cut-off points for joint hypermobility, profession of assessor, total at each Beighton score and sex-specific totals at each Beighton score). Where studies only described a pooled score population (e.g. ≥ 4 Beighton Score), these studies were grouped accordingly within only that cut-off score group. General and sex-specific prevalence were calculated for Beighton scores ≥ 4, ≥ 5, ≥ 6 and ≥ 7 with corresponding standard errors for each world region (i.e. East Europe/Russia, Middle East, North America, Oceania, South America, South/South East Asia and Western Europe). Statistical heterogeneity was quantified according to *I*^2^ statistic and formed the bases for the application of fixed or random effect models. *I*^2^ was > 50% in all analyses; hence, only random effect models were constructed. The PRISMA flow diagram was made using PRISMA2020 (R Package and Shiny app). All effects models were constructed on general and sex-specific prevalence, with sex sub-analysis. Differences between world regions and age groups were assessed by Mann-Witney *U* testing. All analyses were performed in Python, and the base code was made available on https://github.com/HR-Data-Supported-Healthcare. *p* values < 0.05 were considered statistically significant.

## Results

A total of 523 articles were identified from searching, and one article was identified through hand searching (Supplementary File 3). There were 125 articles assessed for eligibility. Of these 125, 28 met the broader inclusion criteria but we were unable to extract or obtain data in a useable form for meta-analysis and therefore excluded. There were a further 60 excluded for other reasons outlined in Supplementary File 3. There were 37 articles reporting on the prevalence or incidence of joint hypermobility at cut-off scores included in meta-analysis from 28,868 children (Table 1). Where data met the assumptions of normality, there were no statistically significant differences between the mean prevalence and any world region for any cut-off score groups (*p* > 0.05).

**Table 1:** Included articles and key demographics of participants where there was a Beighton score of 4 or greater provided

First author, year of publication	Country/world region	Profession of assessor	N (total)	Female n (% of total)	Male n (% of total)	Age (mean, SD or *median, IRQ)* (years)	Age range (years)	Beighton Score	n (%) with corresponding Beighton Score	Female n (% of total females) with corresponding Beighton score	Male n (% of total males) corresponding Beighton score
Abujam, 2014 [14]	India/SSE Asia	Medical	1838	742 (40.4)	1096 (59.6)	11.5 (2.9)	6–17	≥ 4	1081 (58.8)	NR	NR
								≥ 5	844 (45.9)		
								≥ 6	816 (44.4)		
Bout-Tabuku, 2014 [38]	United States of America (USA) (N America)	Medical practitioner and other trained assessors	51	32 (62.7)	19 (37.3)	*14.5 (NR)*	10.2–17.9	≥ 4	10 (19.6)	NR	NR
								≥ 6	2 (4.8)		
Clinch, 2011 [3]	United Kingdom (UK) (W Europe)	Trained assessors, profession not described	6022	3061 (50.8)	2961 (49.2)	13.8 (NR)	13–14	≥ 4	1156 (19.2)	842 (27.5)	314 (10.6)
								≥ 6	252 (4.2)	214 (7.0)	38 (1.3)
Czaprowski, 2015 [37]	Poland (E Europe/Russia)	Profession not described	249	136 (54.6)	113 (45.4)	11.8 (0.8)	10–13	≥ 4	NR	NR	19 (16.8)
								≥ 5	NR	18 (13.2)	NR
deBoer, 2015 [39]	The Netherlands (W Europe))	Physiotherapy	249	138 (55.4)	111 (45.6)	5.7 (0.2)	NR	≥ 4	85 (34.1)	59 (42.8)	26 (23.4)
								≥ 5	56 (22.5)	41 (29.7)	15 (13.5)
								≥ 6	39 (15.7)	29 (21.0)	10 (9.0)
Dhuri 2016 [15]	India ( SSE Asia)	Profession not described	74	37 (50%)	37 (50%)	12.6 (NR)	8–14	≥ 4	10 (13.5)	6 (16.2)	4 (10.8)
Gocentas, 2016 [4]	Lithuania (E Europe/Russia)	Profession not described	778	NR	NR	14.02 (2.11)	10–18	≥ 4	149 (19.2)	NR	NR
								≥ 5	74 (9.5)		
								≥ 6	44 (5.7)		
								≥ 7	2 (0.4)		
Graf, 2019 [16]	Germany (West Europe)	Medical and dental	970	489 (50.4)	481 (49.6)	13.1 (2.1)	10–18	≥ 4	206 (21.2)	132 (27.0)	74 (15.4)
								≥ 5	91 (9.4)	68 (13.9)	23 (4.8)
								≥ 6	49 (5.1)	35 (7.2)	14 (2.9)
								≥ 7	19 (2.0)	17 (3.5)	2 (0.4)
Gurler, 2022 [32]	Turkey (Middle East)	Physiotherapist	33	16 (48.5)	17 (51.5)	11.0 (3.8)	NR	≥ 6	6 (18.7)	NR	NR
Hasija, 2008 [29]	India (SSE Asia)	Medical	656	NR	NR	NR	3–13	≥ 4	435 (66.3)	NR	NR
Jansson, 2004 [2]	Sweden (W Europe)	Profession not described	1845	895 (45.5)	950 (51.4)	12 (3)	NR	≥ 4	778 (42.2)	415 (46.4)	236 (24.8)
								≥ 5	349 (18.9)	233 (26.0)	116 (12.2)
								≥ 6	270 (14.6)	177 (19.8)	93 (9.8)
								≥ 7	106 (5.7)	78 (8.7)	28 (2.2)
Juul-Kristensen, 2009 [40]	Denmark (W Europe)	Trained assessors, profession not described	349	160 (45.8)	189 (54.2)	8.4 (0.52)	NR	≥ 4	101 (28.9)	53 (15.2)	48 (25.4)
								≥ 5	65 (18.6)	34 (9.7)	48 (28.9)
								≥ 6	33 (9.5)	15 (4.3)	18 (9.5)
Lamari, 2005 [17]	Brazil (S America)	Profession not described	1120	586 (52.3)	534 (47.7)	NR	4–7	≥ 4	723 (64.6)	403 (36.0)	320 (59.9)
Leone, 2009 [41]	Italy (W Europe)	Profession not described	1046	516 (49.3)	530 (50.7)	10.8 (NR)	7–15	≥ 4	370 (35.3)	207 (19.8)	163 (30.8)
								≥ 5	232 (22.2)	134 (12.8)	98 (18.5)
								≥ 6	158 (15.1)	91 (8.7)	67 (12.6)
Longworth, 2014 [44]	Australia (Oceania)	Trained assessors, profession not described	30	30 (100)	0 (0)	12 (2.5)	9–16	≥ 5	1 (3.3)	1 (33.3)	-
McCormack, 2004 [42]	UK (W Europe)	Physiotherapy	36	21 (58.3)	15 (42.7)	16.9 (NR)	15.8–18.8	≥ 4	16 (44.4)	13 (36.1)	3 (20)
Mikkelsson, 1996 [33]	Finland (W Europe)	Nursing	1637	835 (51.0)	802 (49.0)	10.8 (1.1)	NR	≥ 4	507 (31.0)	276 (16.9)	231 (28.8)
								≥ 5	228 (13.9)	134 (8.2)	94 (11.7)
								≥ 6	127 (7.8)	70 (4.3)	57 (7.1)
Moore, 2019 [34]	Ireland (W Europe)	Physiotherapy	41	19 (46.3)	22 (53.7)	10.7 (3.6)	6.0–12.0	≥ 4	17 (41.5)	NR	NR
								≥ 5	8 (19.5)		
Morris, 2017 [18]	Australia (Oceania)	Trained assessors, profession not described	1584	769 (48.5)	815 (54.5)	14.1 (NR)	14	≥ 4	760 (48.0)	466 (29.4)	294 (36.1)
								≥ 6	295 (18.6)	201 (12.9)	94 (11.5)
Noormohammadpour, 2019 [19]	Iran (Middle East)	Profession not described	372	NR	NR	15.8 (0.9)	13.0–19.0	≥ 4	59 (15.9)	NR	NR
Qureshi, 2010 [30]	Pakistan (SSE Asia)	Medical	872	398 (45.6)	474 (54.4)	12.85 (3.9)	4–18	≥ 4	323 (37.0)	136 (15.6)	187 (39.5)
Qvindesland, 1999 [20]	Iceland (W Europe)	Nursing	267	143 (53.6)	124 (46.4)	12 (NR)	NR	≥ 4	74 (27.7)	58 (21.7)	16 (12.9)
								≥ 6	26 (9.7)	20 (7.5)	6 (4.3)
Reilly, 2008 [21]	Australia (Oceania)	Profession not described	41	18 (43.9)	23 (56.1)	12.17 (2.57)	8–17.6	≥ 4	8 (19.5)	7 (38.9)	1 (4.3
								≥ 5	5 (12.2)	5 (27.8)	-
								≥ 6	4 (8.8)	4 (22.2)	-
Remvig, 2011 [35]	Denmark (W Europe)	Trained, assessor profession not described	315	159 (50.5)	156 (49.5)	10.1 (0.35)	10	≥ 4	112 (35.6)	60 (37.7)	52 (33.3)
								≥ 5	53 (16.8)	31 (19.5)	22 (14.1)
								≥ 6	35 (11.1)	19 (11.9)	16 (10.3)
Rikken-Bultman, 1997 [31]	Netherlands (W Europe)	Trained assessors, profession not described	910	449 (49.3)	461 (50.7)	NR	4–17	≥ 4	127 (14.0)	85 (18.9)	42 (9.1)
Sabui, 2016 [22]	India (SSE Asia)	Trained assessors, profession not described	3608	1570 (43.5)	2038 (56.5)	NR	3–12	≥ 6	94 (2.6)	54 (3.4)	40 (2.0)
Sanjay, 2013 [23]	India (SSE Asia)	Medical	420	168 (40.0)	252 (60)	NR	6–12	≥ 4	144 (34.3)	NR	NR
Saps, 2018 [24]	Colombia (S America)	Profession not described	136	87 (64.0)	49 (36)	13.4 (2.7)	8–18	≥ 4	38 (27.9)	NR	NR
Seckin, 2005 [25]	Turkey (Middle East)	Profession not described	861	433 (50.3)	428 (49.7)	15.36 (1.1143)	13–19	≥ 4	101 (11.7)	NR	NR
								≥ 5	39 (4.5)		
								≥ 6	24 (2.8)		
								≥ 7	4 (0.5)		
Shulman, 2020 [28]	USA (N America)	Trained assessors, profession not described	69	35 (50.7)	34 (49.3)	9.6 (1.5)	NR	≥ 4	25 (36.2)	NR	NR
								≥ 6	6 (8.7)		
Singh, 2017 [1]	Australia (Oceania)	Profession not described	204	102 (50.0)	102 (50.0)	NR	3–13	≥ 4	49 (47.1)	28 (53.8)	21 (43.4)
								≥ 5	15 (7.4)	11 (10.8)	4 (3.9)
								≥ 6	8 (3.9)	8 (7.8)	-
								≥ 7	3 (1.5)	3 (2.9)	-
Smits-Engelsman, 2011 [5]	Netherlands (W Europe)	Physiotherapy with additional training	551	293 (53.1)	258 (46.8)	8.8 (NR)	6–12	≥ 4	299 (58.5)	NR	NR
								≥ 5	196 (38.4)		
								≥ 6	127 (24.8)		
								≥ 7	50 (9.8)		
Sperotto, 2014 [27]	Italy (W Europe)	Medical	289	143 (49.5)	146 (50.6)	10.8 (NR)	8–13	≥ 4	117 (40.5)	NR	NR
Westling, 1990 [43]	Sweden (W Europe)	Profession not described	193	96 (49.7)	97 (50.3)	17 (NR)	NR	≥ 4	47 (24.4)	27 (14.0)	20 (20.6)
								≥ 5	24 (12.4)	21 10.9)	3 (12.4)
Wright, 2020 [45]	Australia (Oceania)	Allied health or exercise therapist	30	12 (40)	18 (60)	7.9 (1.6	6.0–11.9	≥ 7	7 (23.3)	NR	NR
Yazgan, 2008 [36]	Turkey (Middle East)	Medical	922	413 (44.8)	509 (55.2)	8.26 (1.1)	5–10	≥ 4	363 (39.4)	NR	NR
								≥ 5	210 (22.8)		
								≥ 6	123 (13.3)		
								≥ 7	58 (6.3)		
Zaleski, 2022 [26]	Poland (E Europe/Russia)	Profession not described	200	102 (51.0)	98 (49.0)	7.8 (5.1–12)	3–18	≥ 4	72 (34.0)	NR	NR
								≥ 7	25 (27.5)		NR

### Risk of bias in studies

Table 2 provides the risk of bias analysis for all studies included. The major concern rated unclear or no was absent descriptions of the qualification of assessors or training assessors completed in order to assess and record the Beighton Score (Tables 1 and 2). This occurred in 19 studies [4, 14–31], potentially impacting the intra or inter-rater reliability. Additionally, there were 14 studies reporting the response rate [1, 3, 4, 14, 18, 22, 24, 27, 29, 32–36], while the other studies were unclear or had a low response rate without adequately described overall responses. The strength of the vast majority of studies was the validity of the measures researchers employed to collect the data, in addition to their description of the participants enabling inclusion as data representative of the community.

**Table 2:** Risk of Bias

First author	Sample Frame appropriate	Appropriate participant sampling	Adequate sample size	Description of participants and setting	Data analysis	Valid methods for condition identification	Reliability of measurement of condition	Appropriate statistical analysis	Adequate response rate, or if low, managed appropriately
Abujam [14]	Yes	No	Yes	No	Yes	Yes	Unclear	Yes	Yes
Bout-Tabuku [38]	No	No	No	Yes	Yes	Yes	Yes	Yes	No
Clinch [3]	Yes	Yes	Yes	Yes	Yes	Yes	Yes	Yes	Yes
Czaprowski [37]	Yes	Yes	Yes	Yes	Yes	Yes	Yes	Yes	No
de Boer [39]	Yes	Yes	Yes	Yes	Yes	Yes	Yes	Yes	No
Dhuri [15]	No	No	No	No	No	Yes	Unclear	No	Unclear
Gocentas [4]	Yes	Yes	Yes	Yes	Yes	Yes	Unclear	No	Yes
Graf [16]	Yes	Unclear	Yes	No	No	No	Unclear	No	Unclear
Gurler [32]	No	No	No	Yes	Yes	Yes	Yes	Yes	Yes
Hasija [29]	No	Unclear	Yes	Yes	Yes	Yes	No	Yes	Yes
Jansson [2]	Yes	Yes	Yes	Yes	Unclear	Yes	Yes	Yes	Unclear
Juul-Kristensen [40]	Yes	Yes	Yes	Yes	Unclear	Yes	Yes	Yes	No
Lamari [17]	Yes	Yes	Yes	Yes	Yes	Yes	Unclear	Yes	Unclear
Leone [41]	No	No	Yes	Yes	Yes	Yes	Yes	Yes	Unclear
Longworth [44]	No	No	No	Yes	Yes	Yes	Yes	Yes	Unclear
McCormack [42]	No	No	No	Yes	Yes	Yes	Yes	Yes	Unclear
Mikkelsson [33]	Yes	Yes	Yes	Yes	Yes	Yes	Yes	Yes	Yes
Moore [34]	No	No	No	Yes	Yes	Yes	Yes	No	Yes
Morris [18]	Yes	Yes	Yes	Yes	Yes	Yes	Unclear	Yes	Yes
Noormohammadpour [19]	No	No	Yes	Yes	Yes	Yes	Unclear	Yes	Unclear
Qureshi [30]	No	No	Yes	No	Yes	Yes	No	Yes	Unclear
Qvindesland [20]	No	Unclear	No	Yes	Yes	Yes	Unclear	No	Unclear
Reilly [21]	No	No	No	Yes	Yes	Yes	Unclear	Yes	Unclear
Remvig [35]	Yes	Yes	Yes	Yes	Yes	Yes	Yes	Yes	Yes
Rikken-Bultman [31]	No	Yes	Yes	Yes	Yes	Yes	Unclear	Yes	Unclear
Sabui [22]	Yes	Yes	Yes	Yes	No	Yes	Unclear	Yes	Yes
Sanjay [23]	No	No	Yes	Yes	Yes	Yes	Unclear	No	Unclear
Saps [24]	No	No	Yes	Yes	No	Yes	Unclear	Yes	Yes
Seckin [25]	Yes	Yes	Yes	Yes	Yes	Yes	Unclear	Yes	Unclear
Shulman [28]	No	No	No	Yes	Yes	Yes	No	Yes	Unclear
Singh [1]	Yes	No	Unclear	Yes	Yes	Yes	Yes	No	Yes
Smits-Engelsman [5]	No	Yes	Yes	No	Yes	Yes	Yes	Yes	Unclear
Sperotto [27]	No	No	Yes	Yes	Yes	Yes	Unclear	No	Yes
Westling [43]	Unclear	Unclear	Yes	Unclear	No	Yes	Yes	Yes	Unclear
Wright [45]	No	No	No	Yes	Yes	Yes	Yes	Yes	Unclear
Yazgan [36]	Yes	Yes	Yes	Yes	Yes	Yes	Yes	Yes	Yes
Zaleski ^26^	No	No	Yes	No	Yes	Yes	Unclear	No	Unclear

### Meta-analysis

#### Data from studies reporting children having generalised joint hypermobility at a Beighton score of ≥ 4

**Fig. 1:**
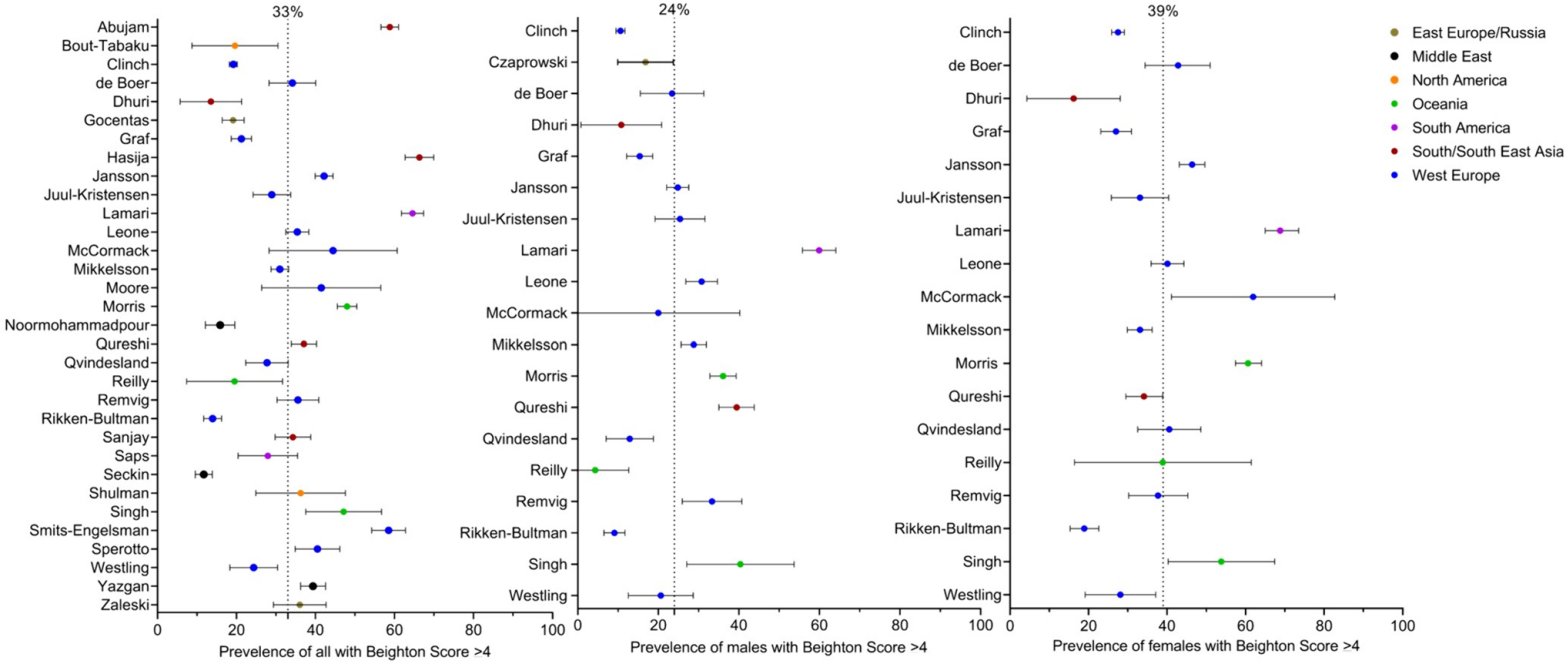
Forest plot random effect models of 33% general prevalence for studies reporting data, 24% for studies reporting male data and 39% for studies reporting female data with a Beighton score of ≥ 4

Prevalence data were extracted from 34 of the 37 studies with data of children rated as having a Beighton score of ≥ 4. The studies were additionally pooled on sex and world region: Eastern Europe and Russia: *n* = 3 [4, 26, 37], Middle East: *n* = 4 [19, 25, 32, 36], North America: *n* = 2 [28, 38], Oceania: *n* = 3 [1, 18, 21], South America: *n* = 2 [17, 24], South and South East Asia: *n* = 5 [14, 15, 23, 29, 30] and Western Europe: *n* = 15 [2, 3, 5, 16, 20, 27, 31, 33–35, 39–43]. In total, 25,060 children were included (reported male = 11,853 and female *n* = 11,441), grand mean age: 12.3 years (Table 1). When considering the general prevalence worldwide from all studies, prevalence varied between 11.7 and 64.6%, with a grand mean prevalence of 33% (Fig. 1). The prevalence of a Beighton score of ≥ 4 varied between 4.3 and 59.9% in males and 16.2 and 68.8% in females. There was a statistically significant mean difference in prevalence between males and females of 14.6% (95% CI = 5.1% to 17.9%, *p* = 0.003).

#### Data from studies reporting children having generalised joint hypermobility at a Beighton score of ≥ 5

**Fig. 2:**
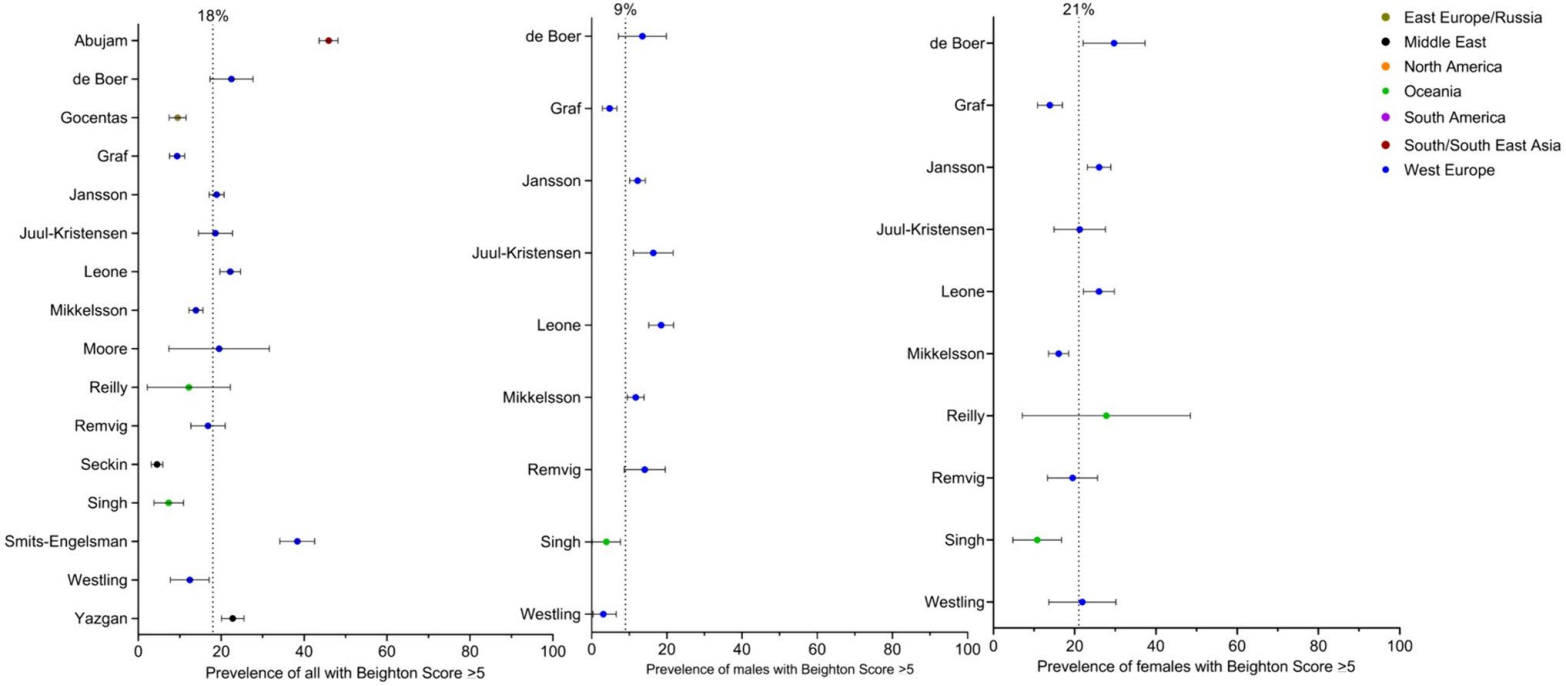
Forest plot random effect models of 18% general prevalence for studies reporting data, 9% for studies reporting male data and 21% for studies reporting female data with a Beighton score of ≥ 5

Prevalence data were extracted from 18 of the 37 studies with data of children rated as having a Beighton score of ≥ 5. The studies were additionally pooled on sex and world region (Eastern Europe and Russia: *n* = 2 [4, 37], Middle East: *n* = 2 [25, 36], Oceania: *n* = 3 [1, 21, 44] South and South East Asia: *n* = 1 [14] and Western Europe *n* = 10 [2, 5, 16, 33–35, 39–41, 43]. In total, 12,079 children were included (reported males *n* = 5867 and females *n* = 5475) with a grand mean age of 11.8 years. When considering the general prevalence worldwide from all studies, prevalence varied between 4.5 and 45.9%, with a grand mean prevalence of 18% (Fig. 2). The prevalence of a Beighton score of ≥ 5 varied between 0 and 18.5% in males and between 3.3 and 29.7% in females. There was a statistically significant mean difference in grand mean prevalence between males and females of 11.5% (95%CI = 5.1% to 17.9%, *p* < 0.001).

#### Data from studies reporting children having generalised joint hypermobility at a Beighton score of ≥ 6

**Fig. 3:**
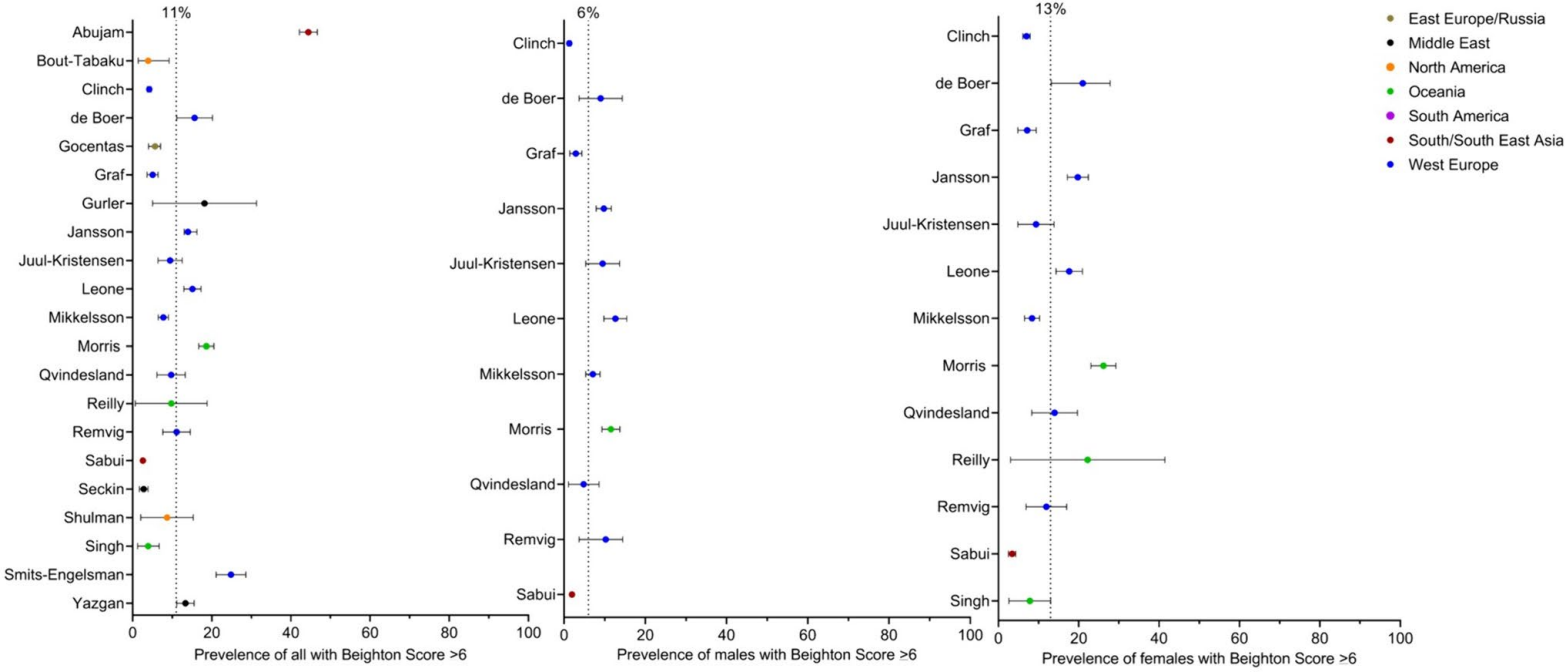
Forest plot random effect models of 11% general prevalence for studies reporting data, 6% for studies reporting male data and 13% for studies reporting female data with a Beighton score of ≥ 6

Prevalence data were extracted from 21 of the 37 studies with data of children rated as having a Beighton score of ≥ 6. The studies were additionally pooled on world region (Eastern Europe and Russia: *n* = 1 [4], Middle East: *n* = 3 [25, 32, 36], North America: *n* = 2 [28, 38], Oceania: *n* = 3 [1, 18, 21], South and South East Asia: *n* = 2 [14, 22] and Western Europe: *n* = 10 [2, 3, 5, 16, 20, 33, 35, 39–41] and sex. In total, 23,200 children were included (reported male *n* = 11,643 and female *n* = 10,867) with a grand mean age: 11.5 years. The prevalence of a Beighton score of ≥ 6 varied between 2.6 and 44.4%, with a grand mean prevalence of 11% (Fig. 3). The prevalence of a Beighton score of ≥ 6 ranged between 0 and 12.6% in males, and in females, it ranged between 3.4 and 7.0%. There was a statistically significant mean difference in grand mean prevalence between males and females of 7.3% (95%CI = 2.2 to 12.4%, *p* = 0.005).

#### Data from studies reporting children having generalised joint hypermobility at a Beighton score of ≥ 7

**Fig. 4:**
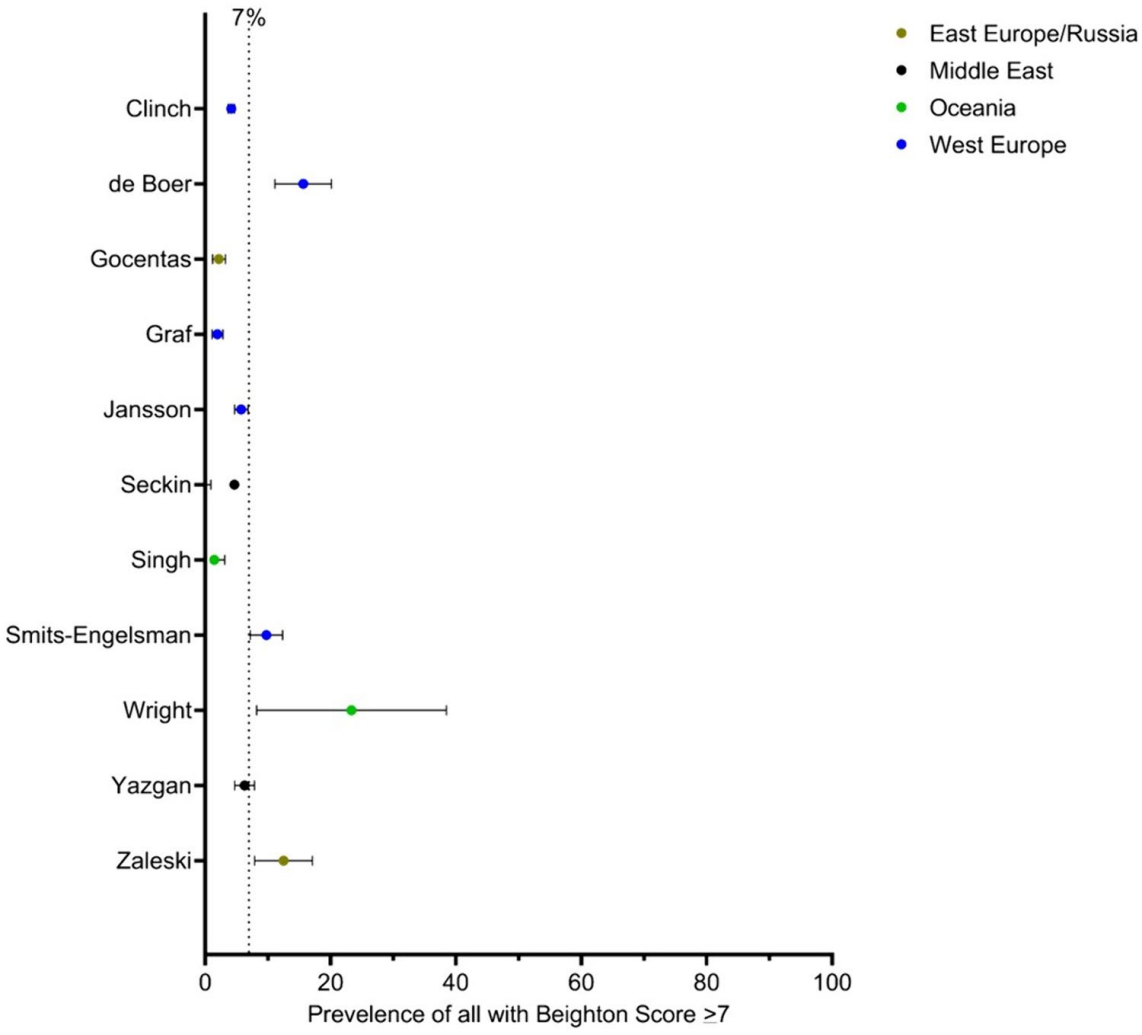
Forest plot random effect models of 7% general prevalence for studies reporting data with a Beighton score of ≥ 7

Prevalence data were extracted from nine of the 37 studies (Fig. 4; Table 1) reporting prevalence for ≥ 7 Beighton scores. The studies were additionally pooled on world region (Eastern Europe and Russia: *n* = 2 [4, 26], Middle East: *n* = 2 [25, 36], Oceania: *n* = 2 [1, 45], Western Europe: *n* = 3 [2, 5, 16] and sex). In total, 6321 children were included (reported male *n* = 3209 and female *n* = 2787), ranging in the ages of 3 to 18 years (grand mean age: 11.3 years). When considering the general prevalence worldwide from all studies, prevalence varied between 0.5 and 23.3%, with a grand mean prevalence of 7% (Fig. 4). The prevalence of a Beighton score of ≥ 7 ranged between 0 and 2.2% in males, and in females, it ranged between 2.9 and 8.7%. Due to the small number of studies reporting prevalence of sexes (*n* = 3), no sub analysis of scores between sexes was performed.

## Discussion

This is the first known study using pooled normative data of Beighton scores from more than 28,868 children around the world. This analysis comprehensively supports rejecting using a Beighton score cut-off of 4 or 5 in paediatric cohorts to identify generalised joint hypermobility. We identified, at a minimum, the working threshold for identifying generalised joint hypermobility in children should be a Beighton score of 6 or more. This is in keeping with the recent Pediatric Joint Hypermobility Diagnostic Framework [8]. Our analysis also suggests a Beighton score of 7 or greater may be appropriate, particularly for females.

Robust screening tools in health care prevent diagnostic and assessment wastage [46]. While the Beighton score has been criticised for its simplicity, it remains an easy-to-learn, reliable and free tool that clinicians can quickly use in both the face-to-face and telehealth formats [47]. Our data-driven approach using worldwide prevalence data identified even with the Beighton cut-off score of 5 or more, that 21% of female children and 9% of male children would be categorised with generalised joint hypermobility. This high prevalence indicates that a score as such is a typical trait of childhood lying within normal variance. Utilising a higher cut-off score of 6 of more is strongly recommended in future clinical and research use.

Clinicians screening children with joint-related or multisystemic concerns should only consider additional screening tools or diagnostic tests related to generalised joint hypermobility disorders, after considering this higher threshold Beighton score. Other comprehensive joint assessment tools, such as the Lower Limb Assessment Scale [48] and the Upper Limb Hypermobility Assessment Tool [49], may then be used to further understand the child’s joint profile at that point in time. Maintaining the higher threshold for identification of generalised joint hypermobility of 6 or more at all paediatric ages requires clinicians acknowledge generalised joint hypermobility status can change over time, one of the key recommendations within the new paediatric hypermobility diagnostic framework [8]. Resisting the status quo of a hypermobility disorder diagnosis based on a lower cut-off score may challenge clinicians and families as a child’s presentation varies over time.

We were unable to fully interrogate our data with respect to age variation, ethnicity outside of world regions, or pubertal status. Limited data availability precluded further sub-analysis of these factors even though some studies identified these variables as important factors impacting joint mobility. We were also unable to analyse the prevalence of the higher Beighton score cut-offs due to limited data reporting. Future studies should ensure, at a minimum, Beighton score data is available based on children’s age, ethnicity and pubertal status. There is also emerging evidence of gender dysphoria in the paediatric hypermobility cohort; future research should consider including reporting both sex assigned at birth and gender identity [50]. One element of bias introduced into the review was limited reporting of assessor training. It is also unknown what impact this had on our results. On-going research has determined the Beighton score assessment inter and intra-rater reliability high in trained assessors [51] and shows promising reliability with skilled observation over telehealth [52]. Improving reporting of assessor training and setting assessment would improve the quality of the evidence in the future.

## Conclusion

This data-driven approach provides clinicians with clear guidance of cut-offs for generalised joint hypermobility in children. These results should give clinicians confidence to reassure families about the wide and typical variation in childhood joint mobility. These results should also provide guidance about a higher threshold for clinicians to consideration if further assessment is required to reduce unnecessary testing, inaccurate or over diagnosis.

### Supplementary Information

Below is the link to the electronic supplementary material.

Supplementary file1 (JPEG 126 KB)

Supplementary file2 (DOCX 15 KB)

Supplementary file3 (PNG 69 KB)

## Data Availability

Data is provided within the manuscript.

## Abbreviations

PRISMA: Preferred Reporting Items for Systematic Reviews and Meta-Analyses
CI: Confidence intervals
PICO: Population, Intervention, Comparison and Outcomes
N/A: Not applicable
