# Supplementary figures and images for: Variability of joint hypermobility in children: a meta-analytic approach to set cut-off scores
Source: Eur J Pediatr. 2024 May 27;183(8):3517–29. doi: 10.1007/s00431-024-05621-4 (PMC11263245; doi:10.1007/s00431-024-05621-4)

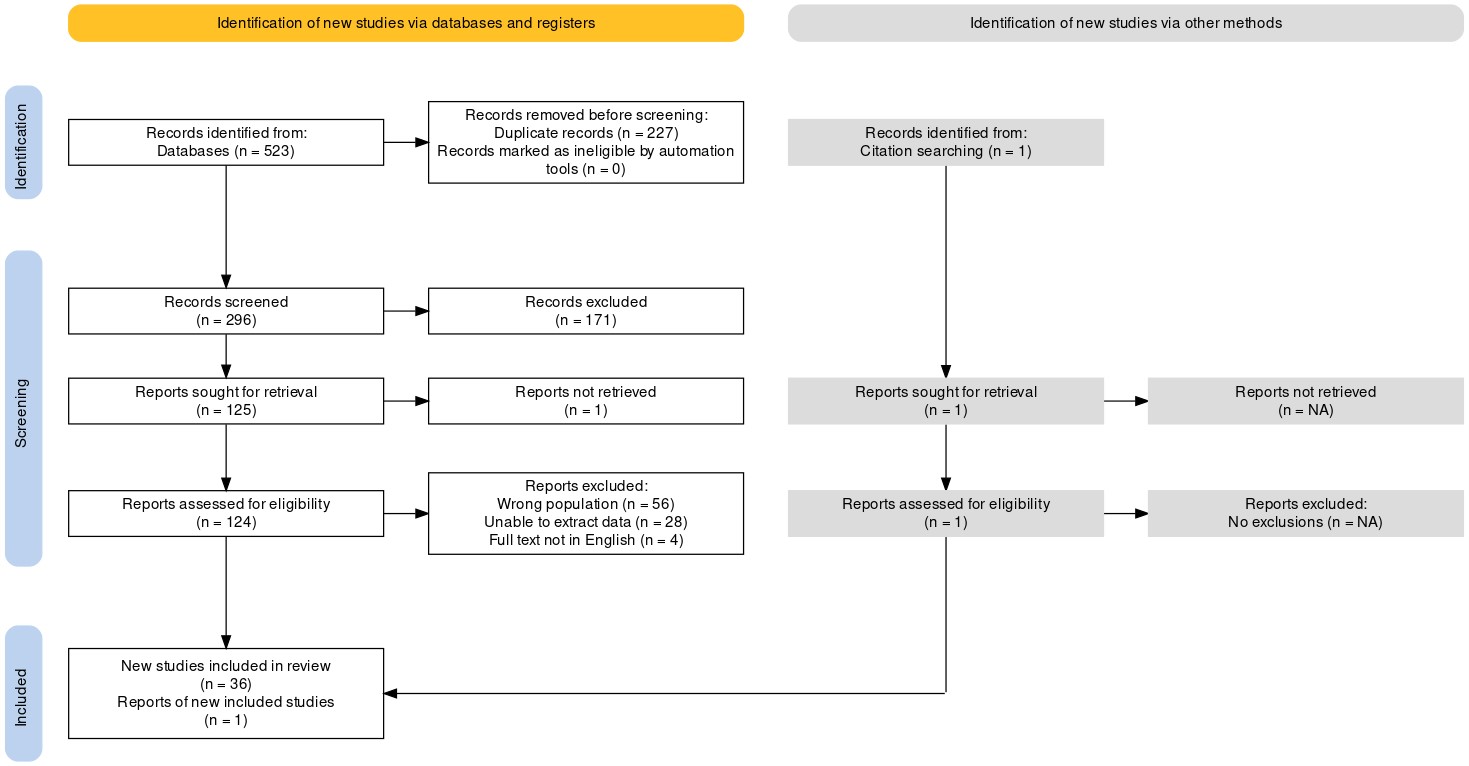

Supplement: Supplementary file 1 — Supplementary file1 (JPEG 126 KB) [file 431_2024_5621_MOESM1_ESM.jpeg]

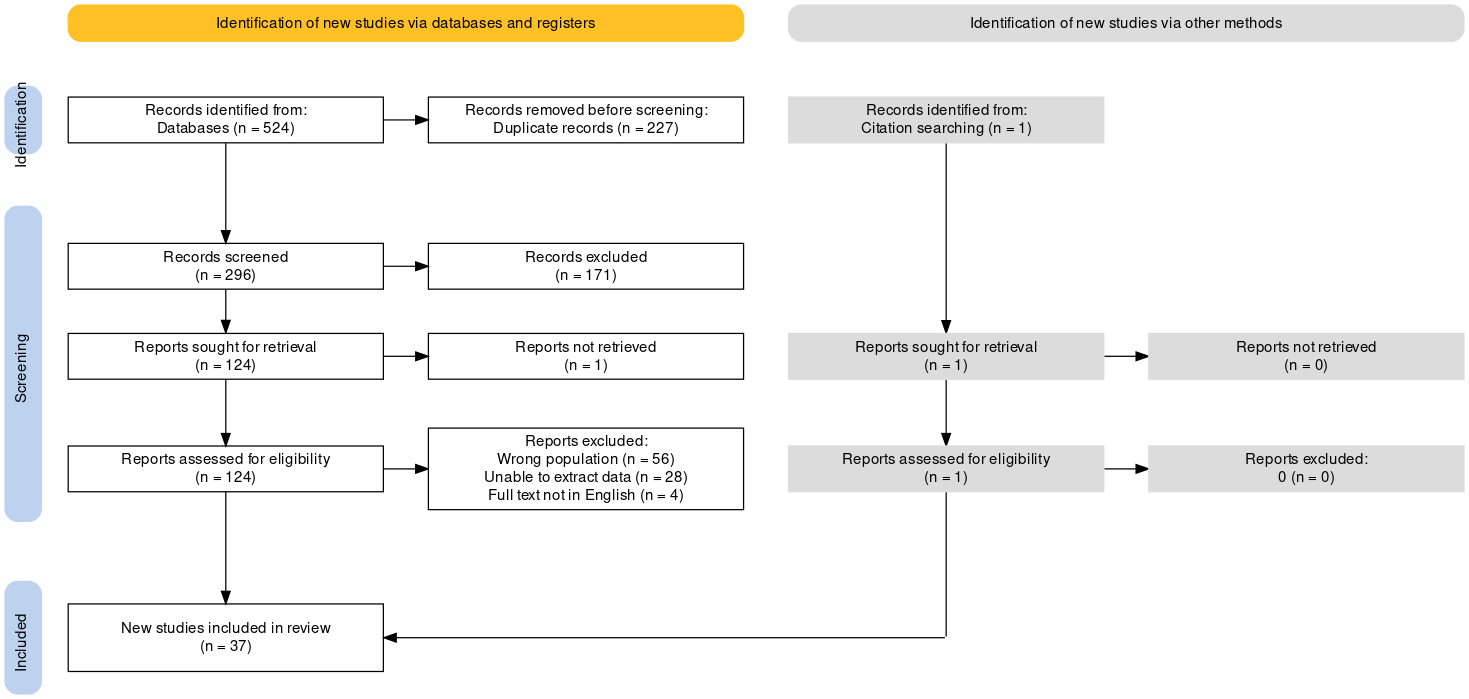

Supplement: Supplementary file 3 — Supplementary file3 (PNG 69 KB) [file 431_2024_5621_MOESM3_ESM.png]
