# Supplementary material for: Variability of joint hypermobility in children: a meta-analytic approach to set cut-off scores
Source: Eur J Pediatr. 2024 May 27;183(8):3517–29. doi: 10.1007/s00431-024-05621-4 (PMC11263245; doi:10.1007/s00431-024-05621-4)
Supplement: Supplementary file 2 — Supplementary file2 (DOCX 15 KB) [file 431_2024_5621_MOESM2_ESM.docx]

**Supplementary File 2: Search strategies**

**Databases:**

AMED (Allied and Complementary Medicine), Ovid MEDLINE, Embase from inception to April 18, 2024

Search terms:

1. child.mp. [mp=ab, hw, ti, tn, ot, dm, mf, dv, kw, nm, fx, kf, ox, px, rx, an, ui, sy]

2. adolescent.mp. [mp=ab, hw, ti, tn, ot, dm, mf, dv, kw, nm, fx, kf, ox, px, rx, an, ui, sy]

3. teenager.mp. [mp=ab, hw, ti, tn, ot, dm, mf, dv, kw, nm, fx, kf, ox, px, rx, an, ui, sy]

4 p$ediatric.mp. [mp=ab, hw, ti, tn, ot, dm, mf, dv, kw, nm, fx, kf, ox, px, rx, an, ui, sy]

5. beighton.mp. [mp=ab, hw, ti, tn, ot, dm, mf, dv, kw, nm, fx, kf, ox, px, rx, an, ui, sy]

6. hypermobility.mp. [mp=ab, hw, ti, tn, ot, dm, mf, dv, kw, nm, fx, kf, ox, px, rx, an, ui, sy]

7. joint instability.mp. [mp=ab, hw, ti, tn, ot, dm, mf, dv, kw, nm, fx, kf, ox, px, rx, an, ui, sy]

8. ligament laxity.mp. [mp=ab, hw, ti, tn, ot, dm, mf, dv, kw, nm, fx, kf, ox, px, rx, an, ui, sy]

9. joint dislocation.mp. [mp=ab, hw, ti, tn, ot, dm, mf, dv, kw, nm, fx, kf, ox, px, rx, an, ui, sy]

10 ehlers danlos.mp. [mp=ab, hw, ti, tn, ot, dm, mf, dv, kw, nm, fx, kf, ox, px, rx, an, ui, sy]

11. 1 or 2 or 3 or 4

12. 6 or 7 or 8 or 9 or 10

13. 11 and 12 and 5

**Database:**

**CINAHL (from inception until 18^th^ April, 2024**

S1 child OR teenage OR adolescents OR p#ediatric

Search modes - Boolean/Phrase

S2 beighton

Search modes - Boolean/Phrase

S3 ehlers danlos OR joint instability OR joint hypermobility OR ligament laxity

Search modes - Boolean/Phrase

S1 AND S2 AND S3

Search modes - Boolean/Phrase
